# Enzyme mechanism prediction: a template matching problem on InterPro signature subspaces

**DOI:** 10.1186/s13104-015-1730-7

**Published:** 2015-12-03

**Authors:** Hamse Y. Mussa, Luna De Ferrari, John B. O. Mitchell

**Affiliations:** EaStCHEM School of Chemistry and Biomedical Sciences Research Complex, University of St Andrews, North Haugh, St Andrews, KY16 9ST Scotland, UK; The Centre for Genomic and Experimental Medicine, Western General Hospital, Crewe Road, Edinburgh, EH4 2XU Scotland, UK

**Keywords:** Enzyme mechanism, InterPro signatures, Nearest-neighbour

## Abstract

**Background:**

We recently reported that one may be able to predict with high accuracy the chemical mechanism of an enzyme by employing a simple pattern recognition approach: a k Nearest Neighbour rule with k = 1 (k_1_NN) and 321 InterPro sequence signatures as enzyme features. The nearest-neighbour rule is known to be highly sensitive to errors in the training data, in particular when the available training dataset is small. This was the case in our previous study, in which our dataset comprised 248 enzymes annotated against 71 enzymatic mechanism labels from the MACiE database. In the current study, we have carefully re-analysed our dataset and prediction results to “explain” why a high variance k_1_NN rule exhibited such remarkable classification performance.

**Results:**

We find that enzymes with different chemical mechanism labels in this dataset reside in barely overlapping subspaces in the feature space defined by the 321 features selected. These features contain the appropriate information needed to accurately classify the enzymatic mechanisms, rendering our classification problem a basic look-up exercise. This observation dovetails with the low misclassification rate we reported.

**Conclusion:**

Our results provide explanations for the “anomaly”—a basic nearest-neighbour algorithm exhibiting remarkable prediction performance for enzymatic mechanism despite the fact that the feature space was large and sparse. Our results also dovetail well with another finding we reported, namely that InterPro signatures are critical for accurate prediction of enzyme mechanism. We also suggest simple rules that might enable one to inductively predict whether a novel enzyme possesses any of our 71 predefined mechanisms.

## Findings

Identification of unknown protein functions is essential for understanding biological processes and beyond [1, 2]. Enzymes are proteins whose function is to catalyse chemical reactions in a living cell. Ascertaining enzymatic mechanisms can have important applications for pharmaceutical and industrial processes in which catalysts are involved [1]. For example, identifying the catalytic mechanism(s) of an enzyme could lead to designing new biocatalysts that give significant cost savings over non-biological alternatives in sectors such as laundry, deodorants, foods and agriculture [1].

Unlike predicting enzymatic functions at the level of the chemical reaction performed [2–4], the problem of predicting by which molecular mechanism a particular enzyme operates has not been well researched [1]. Two of us, De Ferrari and Mitchell, have recently looked into this question. In that work, we utilised a pattern recognition approach to predict chemical mechanisms from enzyme sequences [1]—to the best of our knowledge, that study was the first attempt to predict enzymatic mechanism in this way.

One notable aspect of that work was the excellent prediction success rate of over 96 % for 248 test enzymes—albeit in a leave-one-out setting—even though the training dataset was small and the simple k Nearest Neighbour rule with k = 1 (k_1_NN) [5, 6] was the algorithm employed for pattern classification. The k_1_NN rule is well known to be highly sensitive to errors in the training set [7], in particular when the training dataset is small [7–9]. For example, the number of training examples required for a k_1_NN rule to achieve high classification or prediction accuracy grows exponentially with the number of irrelevant features (noise) [7, 9].

In the light of the “anomaly” described above, we have re-analysed that mechanism dataset and our previous classification results—mainly to understand and explain, if possible, the high prediction success rate achieved.

In the following section, we briefly describe our previous work. The “Results” section presents our new findings, and the final section gives our concluding remarks.

To our knowledge, our study was the first attempt at bulk prediction of enzymatic mechanism from protein sequence [1]. The predictive model was an empirical and observational model [10] based on the concept of pattern classification.

Formally, a pattern classification problem deals with the optimal assignment of an object to one of *J* predefined classes, categories or labels, $\Omega = \left\{ {\omega_{1} ,\omega_{2} , \ldots ,\omega_{J} } \right\}$, whereby it is assumed that the object is adequately characterized by *L* features, *x*_*i*_ with *i* = 1, 2, …, *L*. Typically, the object is represented by an *L*-dimensional vector *x*, whose elements (*x*_1_, *x*_2_  …, *x*_*i*_) are discriminatory features that ideally can identify the object with a low misclassification error rate. In this regard, the classification task is equivalent to establishing a mapping

1 $$f:\chi \to \Omega$$

from the feature space* χ* into the class space Ω, such that $x \in \chi$ is assigned to its appropriate class label $\omega_{j} \in \Omega$, where *j* = 1, 2, …, *J*. Each point in the class space has a corresponding region(s) or subspace(s) in the feature space defined by the *L* features.

In our previous study, the feature *x*_*i*_ denotes absence (0) or presence (1) of an InterPro signature for an enzyme sequence, i.e., $x_{i} = \{ 0,1\}$. In other words, χ was a binary feature space $\chi = \left\{ {0,1} \right\}^{L}$. The class space Ω comprised *J* discrete points each representing one of the enzyme mechanism labels *ω*_*j*_, extracted from Version 3.0 of the MACiE (Mechanism, Annotation and Classification in Enzymes) database [11–13].

The mapping algorithm was the simple k_1_NN classifier. This algorithm can be basically viewed as a dictionary search [14]. That is to say, all the data points allotted for training are stored in a memory (a dictionary in χ), and a test data point is classified to the class label or labels $\omega_{j}$ of the closest point in the dictionary, i.e., in χ. The specific implementation used in our calculations was Mulan’s BRKNN algorithm [5, 15].

Generally speaking, the integration process carried out by InterPro’s curators removes many of the redundant signature matches that might otherwise occur. This results in a relatively small number of InterPro signatures being present for the typical sequence in this dataset. Thus, the squared nearest neighbour distance often takes small integer values, and it is common to find plural nearest neighbours an equal distance away. In this case, the label (or label set) most common amongst the ring of nearest neighbours is assigned.

The mechanism dataset consists of 248 enzymes annotated against 71 MACiE labels, where each enzyme is represented by 321 InterPro signatures—i.e., *L* and *J* are 321 and 71, respectively. We employed a leave-one-out validation scheme: 247 of the enzymes whose mechanisms were known were utilised as a “dictionary” and the mechanism(s) of the one remaining enzyme was predicted, this processes being repeated 248 times. The simple pattern recognition approach yielded an excellent prediction success rate of over 96 % for the 248 test enzymes.

## Methods

In the present work, we are not directly concerned with the question of defining enzyme mechanisms; instead, we just use the mechanism dataset. We focus on finding the reasons why the k_1_NN rule gave us such good classification results for this small dataset, its size being limited by the considerable experimental effort required to characterise enzyme mechanisms.

While directly visualising the 321 dimensional feature space $\chi = \left\{ {0,1} \right\}^{L = 321}$ would be impossible, we were able to go through the dataset manually. The mechanism dataset was represented by a 248-by-323 matrix whose rows were the 248 enzymes, and the first and last columns contained the enzyme names (the enzyme sequence’s UniProt accession number) and their associated mechanism class labels, respectively. The remaining 321 columns denoted the 321 InterPro signature features.

We systematically swapped the 321 columns containing the InterPro signature features while keeping the rows and the first and last columns of the matrix fixed.

## Results

**Fig. 1:**
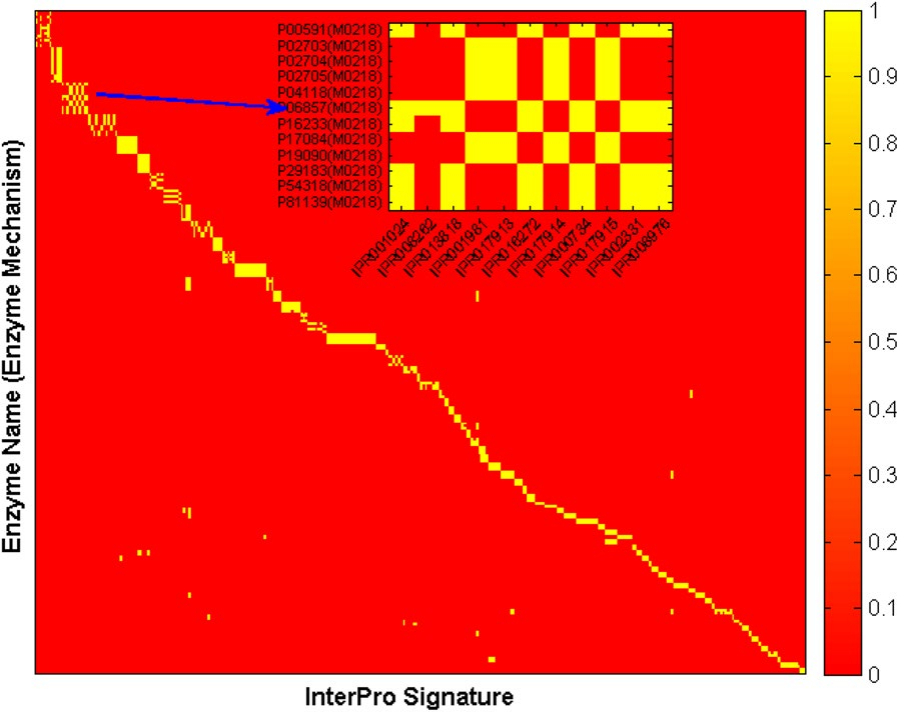
Heatmap of our data matrix. The *horizontal axis* denotes InterPro signatures, whereas the *vertical axis* represents the enzyme sequence’s UniProt accession number and the corresponding MACiE enzymatic mechanism labels of the form M0123. The *yellow colour* signifies that feature *x*_*i*_ (InterPro signature) is present for the enzyme, while the *red colour* indicates that *x*_*i*_ is absent for this enzyme. Enzymes that possess the same MACiE mechanism label reside in a subspace of the feature space *χ*, which barely overlaps with other subspaces associated with other mechanisms. The* inset* depicts the heatmap for the dataset matrix corresponding to the InterPro signatures and names of enzymes with the MACiE enzymatic mechanism label M0218

After a number of iterations, we ended up with a block diagonal version of the original data matrix, see Fig. 1. The figure, a heat map of the data matrix, seems to explain why k_1_NN yielded the excellent classification results [1]. In the figure, the abscissa denotes InterPro signatures, whereas the vertical axis represents the enzyme sequence’s UniProt accession number and the corresponding MACiE enzymatic mechanism labels of the form M0123. The colour yellow signifies that feature *x*_*i*_ (InterPro signature) is present for the enzyme, while the red colour indicates that feature *x*_*i*_ is absent for the enzyme.

According to Fig. 1, the 321 InterPro signatures are highly discriminating features. Enzymes that possess the same enzymatic mechanism $\omega_{j}$ reside in a subspace (region) in $\chi = \left\{ {0,1} \right\}^{L = 321}$ which barely overlaps with neighbouring regions. The inset in Fig. 1 depicts the heatmap of the portion of the dataset that corresponds to the enzymes (and their InterPro signature features) that have MACiE enzymatic mechanism label M0218, i.e. $\omega_{j} = M0218$. Note that a subspace for a given mechanism can be a composite (union) of non-overlapping “sub-subspaces”. The sharing of the M0218 label by two separate non-homologous sequences illustrates the presence of two distinct proteins, firstly pancreatic lipase and secondly colipase, in the reactive complex.

Out of our 71 regions, only the two regions representing enzymes with MACiE mechanisms $\omega_{j = 30} = M0348$ and $\omega_{j = 35} = M0269$ completely overlap. The same four InterPro signature features represent the enzymes that show mechanisms M0348 and M0269, highlighted in red in Table 1.

**Table 1:** Enzymatic MACiE mechanism labels *w*_*j*_ and the number of enzymes reported to possess this mechanism *w*_*j*_

*w*_*j*_	Number of enzymes
M0346	3
M0070	3
M0118	2
M0206	3
M0034	3
M0033	2
M0235	3
M0051	5
M0312	4
M0069	2
M0050	4
M0123	2
M0248	3
M0202	2
M0007	5
M0171	3
M0255	2
M0336	2
M0117	2
M0006	4
M0131	2
M0212	6
M0017	3
M0326	7
M0218	12
M0078	3
M0314	4
M0324	13
M0175	4
*M0348*	2
M0045	5
M0003	3
M0147	7
M0121	2
*M0269*	2
M0253	3
M0026	3
M0188	5
M0130	4
M0159	2
M0213	4
M0249	2
M0055	3
M0272	2
M0122	2
M0060	2
M0148	2
M0303	2
M0029	2
M0071	3
M0099	6
M0126	6
M0262	2
M0177	14
M0013	4
M0021	2
M0015	2
M0228	6
M0058	2
M0211	2
M0309	2
M0154	2
M0244	2
M0209	2
M0270	3
M0063	4
M0328	2
M0039	2
M0252	3
M0036	2
M0080	2

Columns 1 and 2 denote enzymatic MACiE labels in our dataset and the number of enzymes reported for each, respectively. M0123, or similar, denotes the enzymatic mechanism’s label in the MACiE database. The two mechanism labels shown in italics are discussed in the main text

We suggest that our block data-matrix could be employed as an enzymatic mechanism prediction tool—a template against which to match novel enzymes to ascertain their potential enzymatic mechanisms in regard to the 71 mechanisms in the mechanism dataset.

In this work, our mechanism dataset was re-analysed to ascertain as to why a simple but high variance classifier yielded such excellent classification results.

We hope that we have provided a reasonable explanation; the mechanism dataset matrix is block diagonal in the feature and class spaces. In other words, the features (almost) uniquely codify the chemical mechanism of a given enzyme.

Based on these observations, we have also made the suggestion that one might be able to utilise the dataset matrix as an enzymatic mechanism prediction tool.

## Abbreviations

MACiE: mechanism, annotation and classification in enzymes
k_1_NN: k(=1) Nearest Neighbour
